# Measuring what matters: case for a national benchmarking framework for neurological disparities research

**DOI:** 10.1016/j.neuros.2026.100075

**Published:** 2026-09-27

**Authors:** Bruce Ovbiagele, Olajide Williams

**Affiliations:** a Department of Neurology, University of California, San Francisco, United States of America; b Department of Neurology, Columbia University, New York, United States of America

**Keywords:** Neurological, Disparities, Research, Equity, Benchmark, Score, Organizations

## Abstract

**Importance:**

Neurological disease disproportionately burdens racial, ethnic, socioeconomic, and geographically underserved populations in the United States (US), yet no standardized, public accountability mechanism exists to track how academic neurology departments invest in and produce research addressing these disparities.

**Objective:**

To present the scientific and organizational-behavior evidence supporting potential development of BEACON (Benchmark for Equity, Access, and Contributions to Outcomes in Neurology), a composite annual scoring framework that ranks US academic neurology departments on the funding, output, quality, and community engagement of their neurological disparities research.

**Evidence review:**

We synthesize epidemiologic evidence of neurological health disparities, documented inequities in National Institutes of Health (NIH) funding allocation, and the sociological and health-services literature demonstrating that public institutional rankings reliably change organizational behavior.

**Findings:**

Multiple independent literatures spanning legal education, hospital quality reporting, and NIH policy converge on the same mechanism: public, comparative, institution-level measurement reliably redirects institutional investment toward the measured domain. No equivalent measurement currently exists for neurological disparities research specifically.

**Conclusions and relevance:**

A composite ranking such as BEACON could be a scientifically grounded, low-cost lever to redirect institutional attention, philanthropic investment, and faculty recruitment toward reducing neurological health disparities in the US.

## Introduction

Neurological disease does not affect all Americans equally. Black adults are roughly 45% more likely than White adults to die of stroke, and age-adjusted stroke death rates among Black adults aged 35 to 64 years are more than double those of their White counterparts [1,2]. Older Black Americans are approximately twice as likely as older White Americans to develop Alzheimer disease or a related dementia, and Hispanic and Latino Americans face roughly 1.5-fold greater risk [1,2]. Racial and ethnic disparities are compounded by a second, distinct axis of neurological disparity rooted in geography and rurality. Neurologist workforce density varies markedly across the country, and geographic access to general and subspecialty neurologic care, including care for multiple sclerosis and other chronic neurologic disease, is substantially lower in rural and micropolitan areas than in metropolitan areas, with additional access barriers for people with disabilities and the uninsured [3]. This gap is most severe in historically underserved rural regions. In Appalachia, for example, spatial access to neurologists remains 25% to 35% lower than in comparable non-Appalachian communities even after adjustment for area-level deprivation, at a time when the regional burden of neurologic disease is rising faster than the local neurology workforce [4]. These are not new observations. What is new is the recognition, formalized in the National Institute of Neurological Disorders and Stroke (NINDS) 2021–2026 Health Equity Strategic Plan, that eliminating these disparities requires deliberate, trackable investment in disparities-focused research, not merely broad calls for “diversity” in trial enrollment [5]. Despite this institutional commitment at the funding-agency level, no comparable accountability structure exists at the level where research is produced, i.e., the academic neurology department. Departments are ranked annually on total NIH funding [6-8], on U.S. News & World Report hospital reputation [9], and on residency program metrics [10], yet no comparable framework measures their contributions to neurological disparities research as a distinct, trackable output.

In this paper, we propose that this measurement gap is itself a barrier to progress, and we present the evidence base for closing it through a recurring composite national ranking of neurological departments in the United States.

## Gap in stated priority and measured investment

The history of the National Institutes of Health (NIH) illustrates why measurement matters independent of stated institutional values. In 2011, Ginther et al. [11] demonstrated that, after controlling for applicant publication record, training, and prior awards, Black principal investigators were substantially less likely to receive NIH R01 funding than otherwise comparable White investigators. A 2019 follow-up analysis found the gap essentially unchanged eight years later and identified that Black investigators’ choice of research topic, which included community-level and population-health topics closely aligned with disparities research, explained a meaningful share of the funding gap, suggesting the scientific community itself under-resources this type of inquiry [12]. A related analysis found evidence of a compounded funding disadvantage for women of color specifically [13]. Moreover, a 2016–2020 portfolio analysis conducted by the National Institute of Neurological Disorders and Stroke (NINDS) as its Health Equity Strategic Plan, was the first systematic attempt to quantify how much of the Institute’s own funding actually supported health-disparities and health-equity research, using a validated coding protocol rather than self-report [14]. The fact that such an analysis was necessary, i.e., that NINDS could not simply query its existing systems for the answer illustrates the disconnect between stated priorities and measured investment. It is evidence that disparities-focused investment is not currently tracked in a form that supports accountability, whether at the funder level or, by extension, at the level of the institutions receiving that funding.

## Absence of measurement as a missed opportunity

The need for public measurement is only half the argument; the corollary is that the absence of routine, public measurement is not a neutral state; it is itself a policy choice with consequences. The Institute of Medicine’s foundational report on racial and ethnic disparities in health care concluded that without systematic data on which populations were and were not being adequately served, health systems could neither detect disparities nor be held accountable for reducing them, and it made routine data collection and monitoring a central, not peripheral, recommendation [15]. A 2024 National Academies follow-up assessing two decades of progress found that federal and health-system infrastructure for monitoring disparities had expanded substantially in the interim, and it treated continued measurement, monitoring, and public reporting as a precondition for further progress rather than an optional supplement to it [16].

Applied to institutional research investment specifically, current absence of any standardized, department-level accounting of neurological disparities research is not merely a data gap; it is a missed lever for the same kind of accountability that has already been shown to redirect institutional behavior in adjacent domains such as hospital quality reporting and NIH funding policy [17-21]. Every year that no such instrument exists is a year in which no department can be publicly recognized, or held accountable, for its investment or return on investment in this area, and the literature reviewed below suggests that this absence of visibility itself dampens the competitive and reputational incentives that would otherwise drive investment toward closing neurological health disparities.

## Why public measurement changes institutional behavior

The premise underlying a national benchmarking framework for neurological disparities research is not merely that measurement is useful for description, it is that public, comparative, institution-level measurement is one of the most reliably documented levers for changing organizational behavior across sectors. This literature is summarized in Table 1.

In the sociology of organizations, Espeland and Sauder’s study of US law schools coined the term “reactivity” to describe how institutions change their actual practices, i.e., admissions criteria, financial-aid allocation, faculty hiring, once a public ranking exists, even when administrators privately dispute the ranking’s validity [17]. Follow-up work demonstrated that this effect is not transient: rankings become embedded in institutional routines and self-perpetuate through internalized disciplinary pressure, a dynamic they explicitly link to Foucauldian mechanisms of surveillance and normalization [18].

Health services research shows the same mechanism operating in medicine specifically. Pope found that hospitals named to “America’s Best Hospitals” specialty rankings experienced measurable increases in patient volume in the ranked specialty, creating a direct financial incentive for continued investment in that domain [19]. Hibbard and colleagues isolated the mechanism further: in a controlled comparison, Wisconsin hospitals whose obstetric quality data were published publicly improved significantly more than hospitals that received identical data privately, i.e., the public visibility of the metric, not the data itself, drove the improvement [20]. A broader systematic review by Fung et al. confirmed this pattern across the public-reporting literature: publication of comparative institutional performance data was consistently associated with quality-improvement activity specifically targeted at the reported measures [21].

Academic medicine already exhibits this reactivity around NIH funding rankings, even in the absence of any formal mandate. The Blue Ridge Institute for Medical Research’s unofficial annual departmental NIH funding rankings are widely tracked, cited in institutional communications, and referenced by chairs and deans in strategic planning despite reflecting only total dollars, with no adjustment for research topic, quality, or societal relevance [22]. If departments already respond to an unweighted funding total, there is strong reason to expect they would respond similarly, and more specifically, to a validated composite metric that isolates neurological disparities research as its own trackable domain.

## The beacon framework

We propose the development of BEACON (Benchmark for Equity, Access, and Contributions to Outcomes in Neurology), a composite annual scoring framework that ranks US academic neurology departments on the funding, output, quality, and community engagement of their neurological disparities research (Fig. 1). BEACON operationalizes the prevailing scientific evidence base into a five-domain composite score (funding, output quantity, output quality, research capacity, and community engagement) normalized by percentile rank across institutions and recalculated annually on a fiscal-year basis (Table 2). The framework draws its funding and output components from NIH RePORTER (https://reporter.nih.gov/) and PubMed/MEDLINE (https://pubmed.ncbi.nlm.nih.gov/), its quality component from the NIH iCite (https://icite.od.nih.gov/analysis) field-normalized citation metrics, and its community-engagement component from a combination of NIH activity-code filtering (https://grants.nih.gov/funding/activity-codes) and structured review of grant and publication text. A parallel efficiency-adjusted score, normalized to core faculty count, is calculated to ensure that smaller departments with concentrated expertise are not structurally disadvantaged relative to larger, better-resourced programs.

This design deliberately mirrors established instruments such as the U.S. News Best Hospitals methodology and the Blue Ridge rankings, which departments and health systems already treat as consequential, on the theory, supported by the reactivity literature above, that a ranking must resemble the instruments institutions already respond to in order to activate the same behavioral mechanism [17,19,22]. Nonetheless, this initial framework prioritizes feasibility and reproducibility. Its domain weights and aggregation methods should be subsequently subjected to empirical validation, stakeholder review, sensitivity analyses, and iterative refinement over time.

## Pretest: establishing the current landscape

Implementing a comparative ranking without first establishing what the current landscape of neurological disparities research looks like risks conflating genuine institutional performance with artifacts of an unvalidated measurement instrument. NINDS’s own experience is instructive here: before the Institute could act on disparities within its own funding portfolio, it first had to conduct a dedicated retrospective analysis of its 2016–2020 grant portfolio simply to establish, descriptively, how much of its existing funding already supported health-disparities and health-equity research, a baseline that did not previously exist in usable form [14]. We recommend BEACON follow the same sequence at the institutional level. Before any public ranking is released, a descriptive baseline landscape analysis cataloguing current NIH funding, publication output, and citation visibility in neurological disparities research across US academic neurology departments over a recent multi-year window should be conducted and shared privately with participating institutions, consistent with evidence that private feedback should precede public reporting to allow data verification and correction before visibility-driven incentives take effect [20]. This baseline step serves two purposes distinct from the ranking itself: it validates the underlying query logic and data pipeline against ground truth before that pipeline is used to publicly compare institutions, and it establishes trend data against which future annual BEACON scores can be meaningfully interpreted, so that a department’s position reflects genuine change over time rather than first-year measurement noise. We propose a four-phase, three-year ramp-up (Table 3), modeled on National Committee for Quality Assurance (NCQA)’s public-comment-driven measure development process (https://www.ncqa.org/about-ncqa/contact-us/public-comments/) and on Hibbard et al.’s finding that private feedback should precede public reporting to allow institutions to identify and correct data errors before any public release [20]. This sequencing is intended to build institutional trust and methodological legitimacy before BEACON’s first public ranking, reducing the risk that early data errors undermine the instrument’s credibility.

## Strategic drivers and partners

Development of BEACON will require governance that combines methodological expertise with meaningful representation from health-equity researchers, academic departments, patients, community partners, professional societies, and relevant funding and data organizations.

Importantly, particular attention should be given to ensuring that institutions and communities historically disadvantaged by conventional academic metrics participate in the framework’s iterative development and execution. Their involvement is essential to preventing BEACON from simply reproducing existing hierarchies under an equity label. BEACON’s credibility and reach will be especially enhanced by these strategic ownerships and partnerships:

- ***Society for Equity Neuroscience:*** the only multidisciplinary organization with a primary mission to address neurological disparities through science [23]. Disparities-specific expertise requires a dedicated governance structure since it might be difficult to embed BEACON solely within a general neurology society’s existing committee structure without subordinating it to broader institutional priorities.
- ***NINDS/NIH:*** as the federal steward of the Health Equity Strategic Plan and the primary data source (NIH RePORTER, NIH iCite) underlying BEACON’s funding and quality domains, NINDS is a natural nonvoting scientific liaison and potential cooperative-agreement funder, though not a governing member, to preserve independence
- ***American Academy of Neurology and American Neurological Association:*** as the field’s principal membership societies, both offer dissemination channels, annual meeting platforms for releasing BEACON results, and access to departmental leadership, without needing to hold methodology-setting authority themselves.
- ***Association of American Medical Colleges:*** as the holder of faculty roster and institutional data needed for BEACON’s efficiency-adjusted (per-faculty) scoring, and as a channel to departmental and dean’s-office stakeholders.
- ***Disease-specific patient advocacy organizations (American Stroke Association, Alzheimer’s Association, Epilepsy Foundation, National Multiple Sclerosis Society)***: as sources of community and patient-representative governance participation, and as validators of BEACON’s community-engagement scoring domain, mirroring the consumer-representative role such organizations play in National Committee for Quality Assurance (NCQA) governance model.
- ***Blue Ridge Institute for Medical Research:*** as the incumbent, informally influential NIH-funding benchmarking effort in academic medicine; a data-sharing or methodological-consultation relationship could ease institutional adoption of BEACON by building on an instrument, which departments already track.
- ***Bibliometric and citation-data providers (NIH iCite; Overton.io for policy citations):*** as the technical infrastructure partners required for BEACON’s output-quality domain.
- ***Philanthropic funders active in health equity*** (e.g., the Robert Wood Johnson Foundation, given its precedent role seeding NCQA, and disease-focused foundations aligned with the patient advocacy partners above): as seed and bridge funders during BEACON’s first three years.

## Limitations and safeguards

The reactivity literature that motivates BEACON also documents this metric’s principal risk. Sauder and Espeland describe how law schools, once ranked, sometimes optimized narrowly for whatever the ranking could detect rather than for the underlying goal the ranking was meant to proxy, a dynamic consistent with Goodhart’s law [18]. Applied to BEACON, this risk could manifest as departments pursuing publication volume or grant count in nominally disparities-adjacent topics without meaningful community engagement or translational impact. The framework’s design attempts to guard against this in three ways: weighting quality and community engagement components alongside raw output and funding; requiring co-occurrence of neurological and disparities-specific terms rather than incidental keyword matches to qualify a grant or publication; and subjecting the top-scoring institutions each year to manual abstract review to confirm disparities reduction was a primary aim rather than a secondary or boilerplate inclusion.

A second key limitation is attribution: multi-site and subcontracted grants complicate institution-level credit assignment, and departmental boundaries for this work vary across institutions (neurology, neurosurgery, public health, and freestanding neuroscience institutes may each house relevant faculty). We recommend that BEACON’s initial release includes a disclosed methodology paper and a public comment period analogous to the process NINDS itself used in developing its Health Equity Strategic Plan, so that the instrument’s validity is established before, rather than after, it begins to influence institutional behavior [5]. Table 4 displays a critical evaluation of anticipated methodological limitations and corresponding literature-based responses during our consideration about development of the BEACON score.

## Potential institutional resistance

The case made above for why BEACON should be created does not address why an individual academic neurology department might reasonably resist its introduction, and a balanced account of the proposal should consider this counter-perspective directly, distinct from the methodological critiques addressed in Table 4.

First, departments may lack the discretionary capacity to respond to a new metric. Disparities-focused research disproportionately depends on the time of a small number of faculty, often those who are themselves underrepresented in medicine, who already carry disproportionate uncompensated service, mentoring, and diversity-related responsibilities beyond their formal research role, a burden well documented in academic medicine as the “minority tax.”[24]. A department cannot simply convert a ranking incentive into new investment if the faculty best positioned to lead disparities-focused work are already stretched thin. Second, the current federal funding environment constrains any argument that departments should redirect discretionary effort toward a new domain. In February 2025, NIH proposed capping reimbursement of facilities-and-administrative costs at 15%, substantially below most institutions’ negotiated rates; although the policy was enjoined by federal courts pending litigation, the accompanying cancellation of thousands of existing NIH awards has already forced hiring freezes and reduced doctoral and postdoctoral admissions at research-intensive academic medical centers [25]. In this climate, a department chair’s central concern may reasonably be sustaining existing research infrastructure rather than building new capacity in a newly scored domain, whatever that domain’s scientific or societal merit. Third, geography itself may constrain participation independent of institutional will. Neurologist workforce density is markedly lower in rural and historically underserved regions such as Appalachia [9,10], and departments situated in or serving these regions may have genuine difficulty recruiting the disparities-focused clinician-investigators that BEACON is designed to reward, through no fault of departmental strategy or intent. Fourth, department size is a structural constraint distinct from institutional priority-setting, smaller neurology department and programs at less-resourced institutions, including many Minority-Serving Institutions and Historically Black Colleges and Universities, may have too few core faculty to build a critical mass of disparities-focused investigators even when institutional will and community need are both present [28].

These are legitimate objections, and BEACON’s design attempts to respond to each rather than dismiss them. The efficiency-adjusted, per-faculty score described above is intended to prevent smaller or resource-constrained departments from being structurally penalized relative to larger programs, and a distinct "Capacity/Breadth" indicator (Table 4) further separates program size from focused excellence. Since BEACON’s funding, output, and quality domains are constructed entirely from publicly available federal and bibliometric data sources rather than from new institutional reporting, generating a department’s score does not itself require new staff time or survey infrastructure, distinguishing BEACON from many other institutional-ranking or accreditation-adjacent exercises and reducing the marginal burden of participation during a period of constrained departmental resources. Finally, the phased three-year ramp-up (Table 3) is intended in part to surface exactly these capacity and resource concerns during the pretest and private-feedback phases, before any public ranking creates competitive pressure, so that BEACON’s governance body can consider structural accommodations, such as a distinct benchmark tier or normalization approach for smaller, rural-serving, or resource-constrained departments, before its first public release.

## Anticipated impact

If the reactivity mechanism documented in law schools and hospitals generalizes to academic neurology, the anticipated effects of publishing BEACON annually, include increased departmental investment in disparities-focused faculty recruitment and retention; greater competitiveness for NINDS Health Equity funding mechanisms, since departments with existing capacity are better positioned to respond to funding announcements; and increased visibility for community-engaged research models that are otherwise difficult to capture in conventional productivity metrics. Critically, because BEACON isolates disparities-focused work as its own scored domain, it creates an incentive structure that a general NIH-funding or citation-impact ranking cannot: it rewards departments specifically for closing gaps in neurological outcomes, not merely for total scientific output.

## Conclusion

Neurological health disparities are well documented, epidemiologically severe, and the subject of an explicit funding-agency strategic priority, yet institution-level accountability for research addressing them remains effectively unmeasured. A substantial, cross-disciplinary evidence base demonstrates that public, comparative institutional rankings reliably redirect organizational behavior toward the domain being measured, in settings ranging from legal education to hospital quality to, most directly relevant here, NIH funding itself [11,17,19-21]. BEACON applies this well-established mechanism to a specific and consequential gap, i.e., which US academic neurology departments are actually investing in closing neurological health disparities, and how much. We argue that developing and publishing this framework is a scientifically justified and comparatively low-cost intervention to accelerate progress toward neurological health equity. Ultimately, the goal should not merely be to create another static academic ranking but to develop a transparent, evolving measurement system that makes disparities-focused research visible, rewards meaningful institutional contribution, and becomes progressively more capable of measuring community partnership, translation, opportunity, and equity impact.

## Figures and Tables

**Fig. 1.**
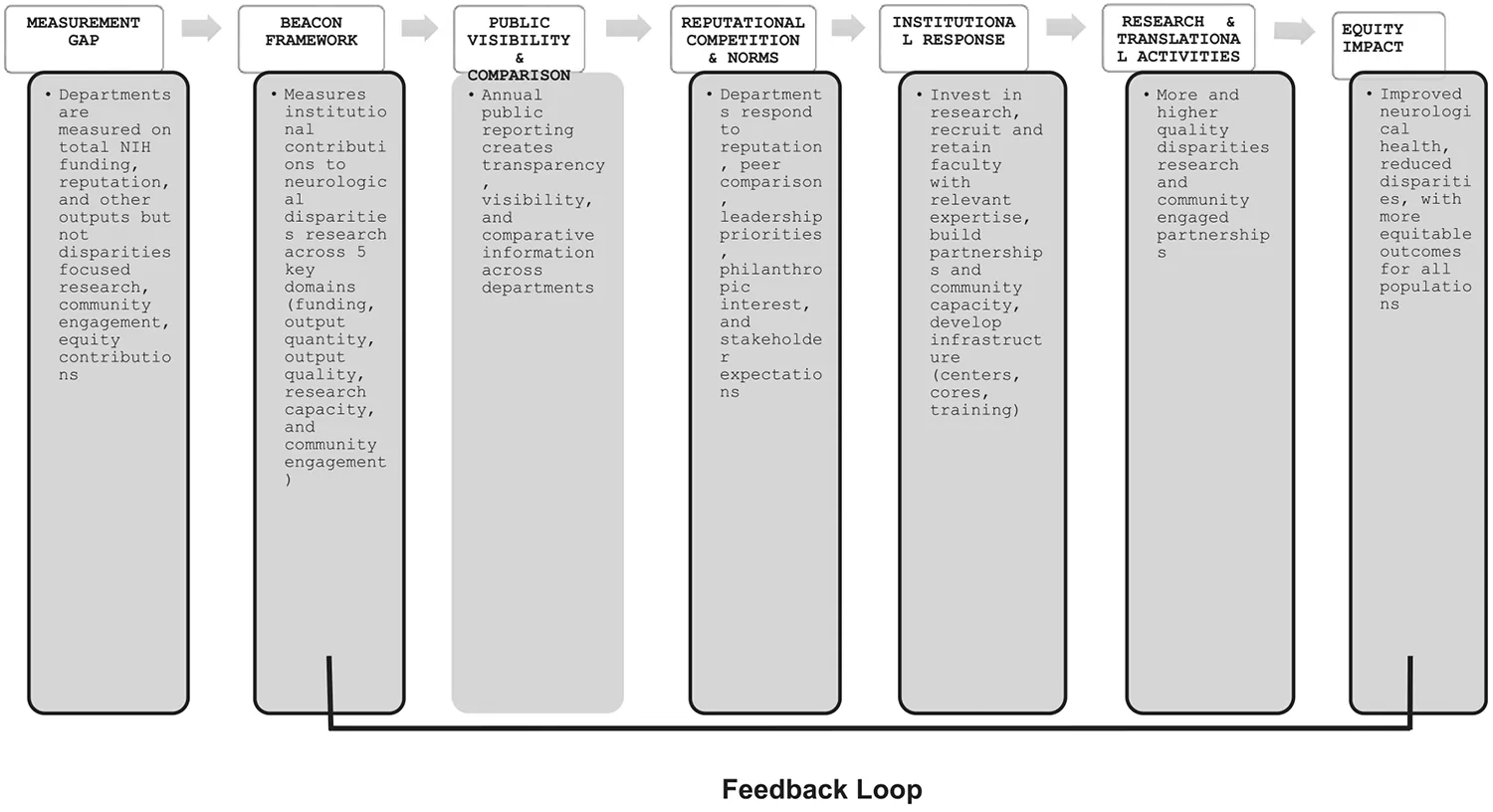
BEACON conceptual framework.

**Table 1:** Evidence that public institutional rankings and benchmarking drive organizational behavior change.

Study / Policy Precedent	Setting	Key Finding Relevant to BEACON
Espeland & Sauder, 2007 [17]	US law schools (US News & World Report rankings)	Coined "reactivity": institutions systematically altered admissions, hiring, and financial-aid practices once a public ranking existed, even when the ranking’s methodology was contested.
Sauder & Espeland, 2009 [18]	US law schools	Rankings became self-perpetuating through internalized, disciplinary pressure on faculty and administrators, producing durable behavior change well beyond initial publication.
Pope, 2009 [19]	US hospitals ("America’s Best Hospitals")	Being named to a specialty ranking measurably shifted patient volume toward ranked hospitals, creating direct financial incentive for institutions to invest in ranked domains.
Hibbard et al., 2003[20]	Wisconsin hospitals (public vs confidential quality reports)	Hospitals whose performance was published publicly improved significantly more than hospitals that received identical data privately, isolating public visibility as the active mechanism of change.
Fung et al., 2008 [21]	Multi-study systematic review of public performance reporting	Across the reviewed literature, publication of comparative institutional performance data was consistently associated with quality-improvement activity targeted at the reported measures.
Ginther et al., 2011[11] and NIH Advisory Committee to the Director response	NIH extramural funding system	Publication of a quantified, institution-level funding disparity prompted a formal NIH working group and 13 policy recommendations, illustrating that a single rigorous quantitative benchmark can mobilize agency-level structural response.
Blue Ridge Institute for Medical Research annual rankings [6]	US academic medical center departments	Departments and deans’ offices actively track and publicize departmental position on these unofficial rankings, demonstrating that clinical-science departments already respond to composite NIH funding benchmarks even without a mandate to do so.

**Table 2:** BEACON composite scoring framework.

Domain	Weight	Representative Metrics	Primary Data Source
Funding	20%	Total annual NIH direct/indirect costs for qualifying disparities-focused neurological grants Number of active awards 3-year funding trend	NIH RePORTER Project API
Output quantity	25%	Peer-reviewed publications per year matching neurological and disparities/equity terms with departmental affiliation First/senior-authored subset	PubMed/MEDLINE via NCBI E-utilities
Output quality	25%	Field-weighted Relative Citation Ratio Journal percentile Policy/guideline citation of departmental work	NIH iCite; Overton.io policy-citation index
Research capacity	10%	Number of distinct NIH-funded principal investigators in this space Presence of disparities-focused T32/R25/K-series training awards Named health-equity center or chair	NIH RePORTER; institutional verification
Community engagement	20%	Active community-engaged research infrastructure awards Disclosed community-based participatory research/community advisory board participation	NIH RePORTER; abstract/publication text review

**Table 3:** Proposed BEACON ramp-up and implementation timeline.

Phase / Timeframe	Milestones
Year 0, Months 1–6 Foundation	Conduct a descriptive baseline landscape analysis cataloguing current National Institutes of Health (NIH) funding, publication output, and citation visibility in neurological disparities research across United States academic neurology departments over a recent multi-year window Assemble a founding group of methodologists, National Institute of Neurological Disorders and Stroke (NINDS) liaison, patient-advocacy representatives, health-equity researchers, community members Secure philanthropic seed grant Finalize methodology white paper
Year 0, Months 6–12 Validation	Open 60-day public comment period on methodology via social media channels (modeled on NCQA’s HEDIS process) Convene external methodology review panel Conduct manual-abstract validation of query logic on a test set of institutions Publish finalized public methodology
Year 1 Pilot	Recruit 15–20 volunteer academic neurology departments spanning department size and NIH funding tier Run BEACON scoring in “private feedback” mode only (mirroring the Hibbard et al. design) so pilot institutions can review and contest their own data before any public release
Year 2 Public Launch	Publish first public BEACON ranking covering all NIH-funded US academic neurology departments Launch public-facing dashboard Open first annual public comment cycle for methodology refinement Begin institutional subscription program
Year 3 and Beyond Steady State	Publish BEACON annually each spring following NIH fiscal-year close Conduct a scheduled 3-year methodology review Expand strategic partnerships and data-sharing agreements Evaluate expansion to related domains (e.g., neurosurgery, child neurology) based on demand

**Table 4:** Critical appraisal of anticipated methodological limitations and literature-based responses.

Limitation	Assessment	Response(s)	Literature
Scoring may reward accumulated institutional wealth and prestige vs. disparities-reduction itself.	This mirrors the well-documented “Matthew effect” in science, where prior advantage predicts future advantage independent of merit, and is empirically visible in Minority Serving Institutions/Historically Black Colleges & Universities federal funding data.	Mitigate this by design (efficiency-adjusted scoring, capped funding weight) but cannot fully escape a dynamic documented across all of science We will publish both Absolute and Efficiency scores side by side rather than a single number, so prestige-driven and disparities-specific performance can be distinguished by the reader.	Merton, 1968 [26,27]; Minority Serving Institutions/Historically Black Colleges & Universities federal funding disparity analyses [28]
Funding may be overweighted in the composite.	A single quantitative proxy should not dominate a multidimensional construct.	Reduce funding’s weight and, more importantly, move toward the multidimensional scorecard, which structurally prevents any one domain from dominating.	DORA, 2013 [29]; Hicks et al., 2015 (Leiden Manifesto) [30]
Double counting: funding, publications, citations, centers, and leadership all reflect one underlying advantage, inflating its apparent weight.	Linearly summing correlated domains overstates the independent contribution of anyone.	Use either a principal-component-adjusted composite that accounts for inter-domain correlation, or preferably, reporting domains as a separate scorecard profile rather than a single weighted sum, consistent with the Leiden Manifesto’s caution against conflating multiple correlated indicators into one score.	Hicks et al., 2015 [30]
Faculty-size normalization omits research full time equivalent, protected time, institutional research expenditures, philanthropy, and Clinical and Translational Science Awards (CTSA) Program infrastructure.	Raw faculty headcount is a weak proxy for actual disparities-research capacity.	Will supplement the efficiency denominator with NSF Higher Education Research and Development (HERD) Survey institutional research-expenditure data and CTSA hub status, both of which are already collected in standardized form and avoid the need for self-reported FTE.	NSF HERD Survey (https://ncses.nsf.gov/surveys/higher-education-research-development/2024)
Efficiency may not equal excellence: a few highly productive investigators could outscore a larger, broader disparities program.	Efficiency and program breadth are different constructs and should not be conflated into one score.	Consistent with the Leiden Manifesto’s principle of measuring performance against an institution’s own mission, we recommend a separate “Capacity/Breadth” indicator distinct from the per-capita Efficiency score, so a focused two-investigator program and a broad multidisciplinary center are not forced onto the same scale.	Hicks et al., 2015 [30]
Prestigious-journal weighting (NEJM, Nature Medicine, Lancet Neurology) compounds publication bias against CBPR, implementation science, and community-focused work.	The San Francisco Declaration on Research Assessment (DORA) explicitly recommends against using journal identity as a proxy for research quality, and the Leiden Manifesto calls for explicitly protecting “excellence in locally relevant research.”	We will remove named-journal weighting entirely from BEACON’s quality domain, relying instead on field-normalized citation impact plus explicit, separately scored credit for Community-Based Participatory Research and implementation-science methodology.	DORA, 2013 [29]; Hicks et al., 2015 [30]
Citation metrics may introduce further bias, favoring established investigators and larger fields.	A cross-sectional study of five high-impact medical journals found articles with women primary or senior authors received significantly fewer citations than comparable articles by men, independent of content, a pattern plausibly extending to author race and institutional prestige.	We will supplement citation-based quality metrics with non-citation indicators (policy and guideline citation) that are less susceptible to network-based citation practices.	Chatterjee & Werner [31]
Scientific influence is not the same as equity impact; publications and citations do not show that disparities in outcomes improved.	A Valid and, at present, only partially solvable limitation.	We could add an aspirational “Equity Impact” domain that links funded disparities research, where feasible, to publicly available population-level outcome-disparity trend data (e.g., Centers for Disease Control and Prevention geographic stroke-mortality disparity tracking), while transparently disclosing that outcome attribution to a specific department’s research remains methodologically difficult and should carry lower confidence than the other domains.	Flynn et al., 2022 [32]
Community engagement might be underweighted.	NIH’s own Research Centers in Minority Institutions (RCMI) program structures Community Engagement as one of only four required program cores thus allowing for effectively a much larger share of programmatic emphasis	We will increase this domain’s weight and/or elevate it to a standalone scorecard domain rather than a minor sub-component of a single composite.	Research Centers in Minority Institutions program core structure [33]; Wallerstein et al., 2020 [34]
Community engagement may be measuring documentation and financing of engagement rather than its authenticity, duration, or reciprocity.	This is why the Community-Based Participatory Research field has spent over two decades building validated partnership-quality instruments rather than relying on grant text or funding alone.	We will incorporate an established, validated partnership-assessment instrument (e.g., the Israel et al. partnership evaluation framework or Wallerstein’s Partnership Data Report tools) with voluntary community-partner co-attestation, rather than inferring engagement quality from abstract text mining alone.	Israel et al., 1998 [35]; Wallerstein et al., 2020 [34]
Collaborative research may be misattributed, with prime-award institutions credited over subcontracting institutions, MSIs, and community partners.	Valid, and consequential: Multiple Principal Investigators are disproportionately relegated to subcontractor roles because of documented, structural capacity constraints in federal grant administration, so a prime-award-only credit rule would compound an existing inequity rather than correct for it.	We will institute a “Partnership Credit” mechanism that allocates partial score to named subaward institutions and community partners identified in NIH RePORTER subproject records, modeled on NIH’s own multiple-PI recognition policy.	Minority Serving Institutions/Historically Black Colleges & Universities capacity-constraint literature [28]
Departmental boundaries create systematic bias, since disparities researchers may sit in public health, social work, nursing, or interdisciplinary centers rather than neurology departments.	A limitation of the underlying methodology	We will score at the level of a defined “neurological disparities research program,” which may span multiple administrative departments within an institution, verified through manual institutional confirmation during the proposed Year 1 pilot rather than relying on department name alone.	Consistent with field-normalization principles in Hicks et al., 2015 [30]
Manual review of only the top 10% of institutions introduces differential, not comparable, verification across the full ranking.	Concentrating scrutiny only at the top both under-verifies lower-ranked institutions and singles out top performers for extra suspicion.	We will adopt NCQA’s Healthcare Effectiveness Data and Information Set (HEDIS) model, in which independent compliance audits apply to all reporting organizations on a standardized schedule, and moving BEACON to stratified random sampling for validation across all score tiers rather than a top-decile-only review.	NCQA HEDIS compliance audit model[36]
A single composite ranking risks becoming a conventional academic prestige ranking filtered for disparities topics; a multidimensional scorecard may better capture true performance.	This echoes the Leiden Manifesto’s core caution against reducing complex performance to one number, and it already has precedent within disparities-research infrastructure specifically: NIH’s own Research Centers in Minority Institutions (RCMI) program evaluation framework was deliberately built as a multidimensional model (capacity, community engagement, research productivity, and outcomes) rather than a single score.	We will ultimately adopt six scored domains: Scientific Contribution Equity Impact Community Partnership Translation/Implementation Capacity Building Opportunity-Adjusted Performance Will retain a single composite only as an initial or optional, clearly labeled summary figure atop the full multidimensional profile.	Hicks et al., 2015 [30]; Research Centers in Minority Institutions evaluation framework [33]
